# Chirality Switching in 1*T*‑TaS_2_ by Highly Charged Ion Irradiation

**DOI:** 10.1021/acs.nanolett.5c04268

**Published:** 2026-02-06

**Authors:** A. Niggas, J. Buck, D. Thima, V. Vojtech, F. Vuković, M. Werl, K. Rossnagel, R. A. Wilhelm

**Affiliations:** † 27259 TU Wien, Institute of Applied Physics, Vienna, 1040, Austria; ‡ 9179 Christian-Albrechts-Universität zu Kiel, Institute of Experimental and Applied Physics, Kiel, 24098, Germany; § 28332 Deutsches Elektronen-Synchrotron DESY, Ruprecht Haensel Laboratory, Hamburg, 22607, Germany

**Keywords:** highly charged ions, ARPES, band structure, chirality, charge density wave, TaS_2_

## Abstract

In layered materials, charge density waves can occur in distinct chiral phases, which can be switched. We use Xe^8+^ ions at a kinetic energy of 22.5 keV to switch the commensurate charge density wave chirality on the nanoscale in 1*T*-TaS_2_ at 50 K. Changes in spectral weight, density of states, and band structure are monitored *in situ* by angle-resolved photoemission spectroscopy. We find that changes in the band structure are most pronounced at the charge density wave gaps and that chirality switches gradually with ion fluence, saturating to near-full handedness reversal at ≳4000 ions/μm^2^. We discuss a scenario for ion-induced chiral switching within the framework of intense, spatially confined electronic excitations, which induce a phase transition and defect-stabilized grain boundaries.

## Graphical abstract

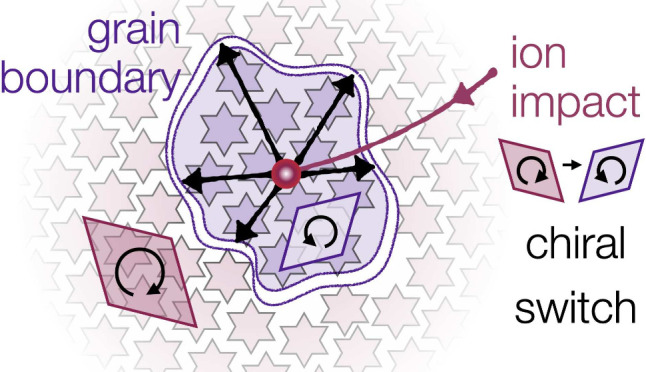

Strong electron correlations in materials are responsible for effects such as magnetism and high-temperature superconductivity.^1–3^ Some van der Waals-layered materials, such as the transition metal dichalcogenide 1*T*-TaS_2_, exhibit electron correlation effects as a side effect of coupled charge density waves (CDW) and periodic lattice distortions at low temperatures.^4^ Specifically, 1*T*-TaS_2_ undergoes a series of CDW phase transitions when cooled until it reaches a Peierls–Mott insulator phase in conjunction with a commensurate CDW (CCDW).^5^ In the CCDW phase, the lattice is reconstructed by the formation of a superlattice of Star of David (SoD) structures in the Ta sublattice (cf. Figure 4).^6^ Interestingly, this SoD superlattice exhibits two-dimensional (2D) chirality.^7–9^

**Figure 1:**
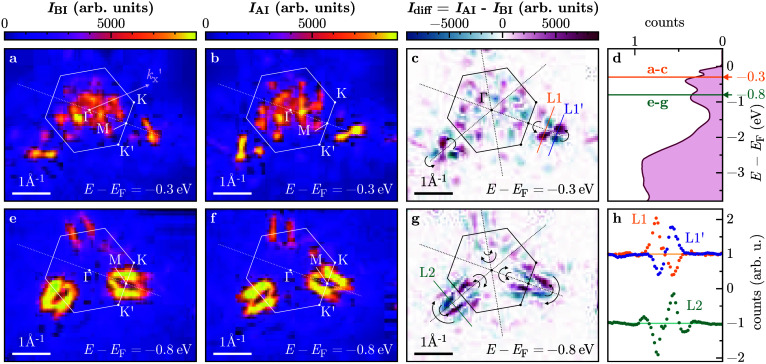
ARPES maps at *E* – *E*_F_ = −0.3 eV (a–c) and *E* – *E*_F_ = −0.8 eV (e–g). The maps (a) and (e) present the intensity recorded before *I*_BI_ and (b) and (f) the intensity after the ion irradiation *I*_AI_. The data in (c) and (g) show the difference *I*_AI_ – *I*_BI_ between the corresponding maps at −0.3 eV and −0.8 eV, respectively. The energies for the maps are indicated as an orange line in (d), together with the energy spectrum of the full angular map recorded before the irradiation. In panels (a–c) and (e–g), the Brillouin zone is indicated with a white/black hexagon and high-symmetry points Γ, *K*, *K′*, and *M* are labeled. A scale bar indicates the *k*-scale (equal for *k*_*x*_ and *k*_*y*_ directions); $\overset{®}{\Gamma M}∼1.1$ Å^–1^ and $\overset{®}{\Gamma K}∼1.2$ Å^–1^. The ΓM symmetry line is indicated as a white/black dashed line. In (h), the line profiles L1 and L1′ as well as L2 are shown as indicated in panels (c) and (g), respectively. Note that the line profiles L1 and L1′ are vertically shifted to +1, and L2 to – 1 to allow for better comparability.

**Figure 2:**
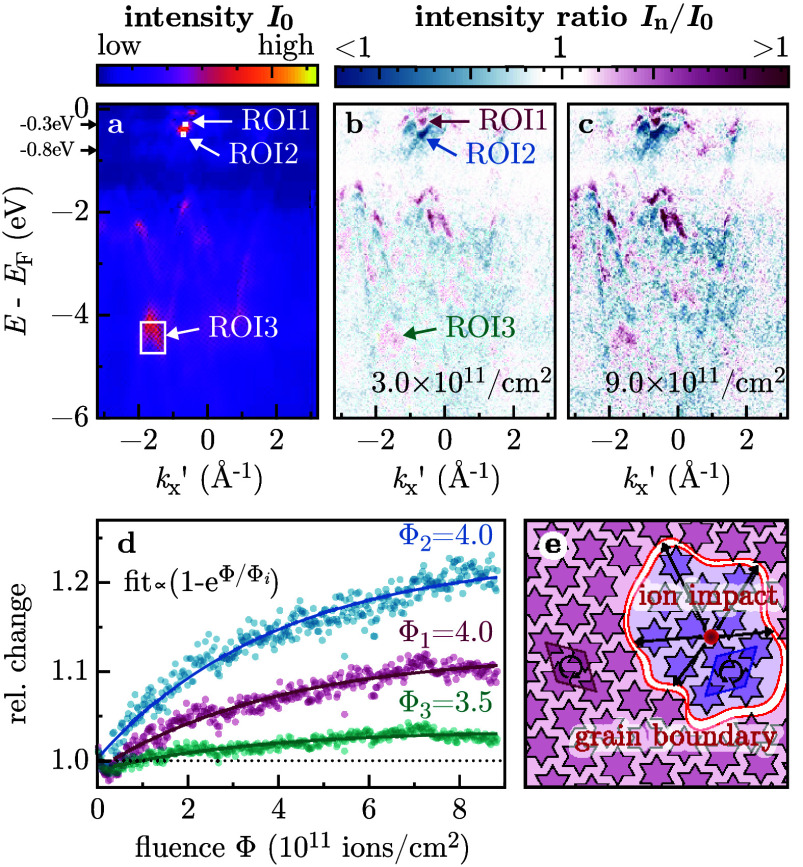
(a) Initial short ARPES map (mean of the first five maps) at the beginning of ion irradiation. Arrows at −0.3 eV and −0.8 eV indicate the energy levels used for the cuts presented in Figure 1. (b, c) Normalized relative change between the initial short ARPES map and selected ones at the indicated ion fluences. The color scale is the difference in the intensity at each pixel. The *k*_*x*_ *′* direction is indicated with respect to the Brillouin zone in Figure 1(a); *k*_*x*_ *′* ∼ 0 corresponds to the Γ point. (d) Photoelectron intensity in the ROIs indicated in (a) and (b) as a function of the applied ion fluence (ROI1 and ROI2: Ta 5d, ROI3: S 3p). The data are fitted by an exponential, and the decay constant is given. (e) Possible chiral domain formed by the ion impact with a defect-stabilized grain boundary.

**Figure 3:**
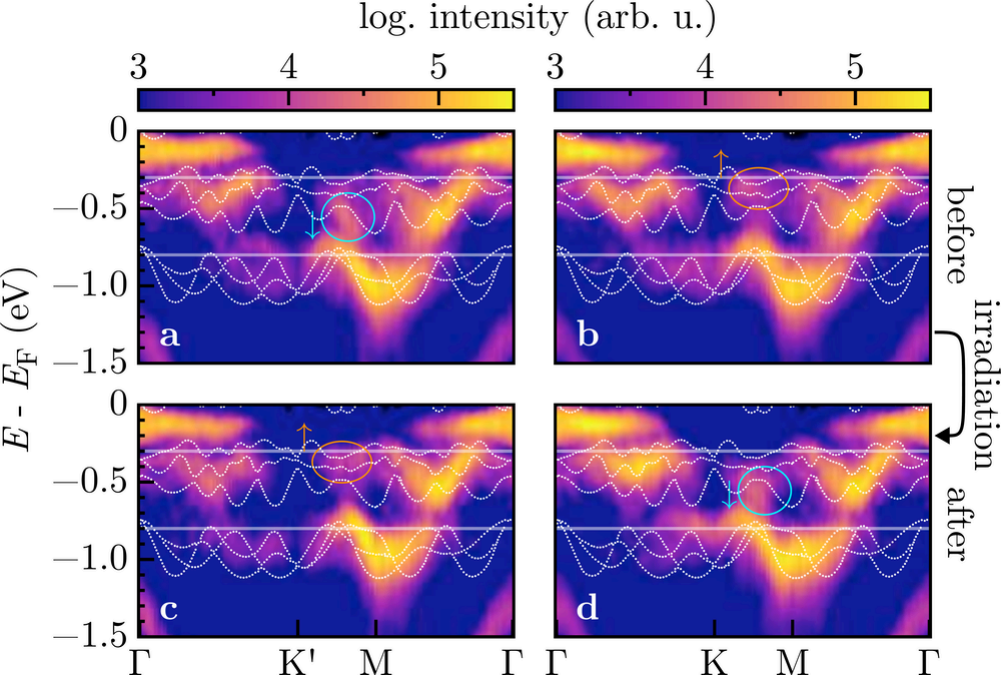
Energy maps before (a, b) and after (c, d) the irradiation for two *k* paths as denoted in Figure 1(a) together with band structure calculations from Ref. 42. (a, c) Γ*K′M*Γ and (b, d) Γ*KM*Γ. White lines indicate the energy levels at −0.3 eV and −0.8 eV used for the energy cuts shown in Figure 1. Circled areas indicate a redistribution of intensity in the Ta 5d bands.

**Figure 4:**
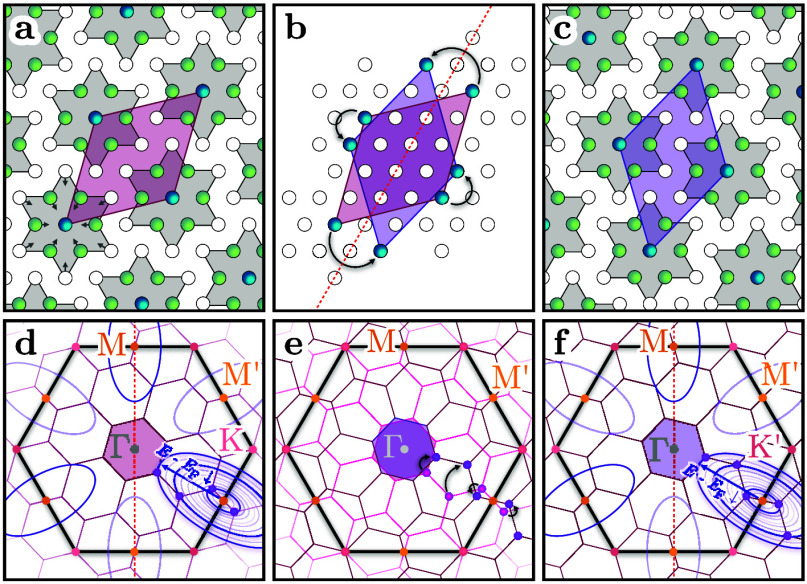
(a) Unit cell of the periodic lattice distortion (red parallelogram) and SoD clusters (gray areas) in the Ta plane. Arrows indicate the displacements of the Ta atoms from their original positions. (b) Change of positions of the SoD centers and correspondingly the rotation of the lattice distortion unit cell (violet line) due to a mirror operation across the Γ*M* line. (c) Final state of the Ta sublattice. (d) Brillouin zones in the normal (black solid lines) and reconstructed (thin red lines) phases. The unreconstructed Ta 5d Fermi surface has elliptical pockets, indicated as violet solid lines. These ellipses appear with increasing size for larger values of *E* – *E*_F_. For specific energies, the ellipses intersect with high symmetry points of the reconstructed Brillouin zone, indicated as violet points. The mirror symmetry is indicated as a red dotted line along the Γ*M* direction. (e) Original (pink) and mirrored (and rotated by 120° for clarity) and (red) Brillouin zones. The intersections are moving in *k*-space. (f) Same as (d) after a mirror operation.

The ability to manipulate correlated electron states in a desired fashion is a powerful tool to tune quantum material properties.^10–12^ Recently, work on the manipulation of the CCDW phase in 1*T*-TaS_2_ and similar materials through the application of strain,^13^ heat,^14^ dopant atoms,^15^ external electric fields,^3,16^ or optical excitations^5,10,17^ received traction. Some of these CCDW engineering strategies work on the nanoscale (e.g., in a scanning tunneling microscope (STM)^18^), while others work on much larger length scales. Similarly, control of the CCDW can be achieved by optical excitations on the ∼10–1000 fs time scale^5,19–22^ with ultrafast lasers (with ∼μm focus), while other methods work in the pico- to nanosecond regime. Here, we introduce a novel approach to switch the 2D chirality of the CCDW in 1*T*-TaS_2_ on both the nanoscale and the femtosecond time scale through irradiation with a low fluence of highly charged ions (HCIs). In particular, we observe changes in the band structure of 1*T*-TaS_2_ previously linked to a reorientation of the surface reconstruction in the CCDW phase, i.e., a rotation of the superstructure Brillouin zone by 27.8°.

Ion beams are a common tool for preparing atomically defined surfaces^23^ and tailoring mechanical surface properties for technological applications.^24^ While heavy ions at kinetic energies of 1–100 keV introduce surface defects by knock-on lattice atom displacements, HCIs additionally strongly interact with the electronic system of the solid.^25,26^ Electronic excitation and deexcitation processes triggered by the ion neutralization may result in structural modifications at the surface.^27,28^ As such, these modifications are essentially confined to the outermost material layers.^29,30^ Slow HCIs also cause the extraction of dozens of electrons from the valence band around the ion impact point.^31^ Some of these electrons are captured by the ion, facilitating its neutralization,^26^ while others are emitted from the surface into a vacuum.^32^ In this study, we use Xe^8+^ ions, storing a potential energy of 417 eV, which is transferred within <3 fs.^33^ We discuss that this energy is released within an area of ≲20 nm in diameter, yielding an average energy density of ∼1.5 eV/nm^2^ = 0.03 mJ/cm^2^ or a power density of ∼10^10^ W/cm^2^, well comparable to typical energy densities applied for optical excitations of the CCDW in 1*T*-TaS_2_.^17^

Here, we report the first *in situ* angle-resolved photoemission spectroscopy (ARPES) that tracks electronic band structure changes induced by ion beams and, in particular, by slow HCIs. Our data show that even at small ion fluences of <4000 ions per μm^2^ the 2D surface chirality can be completely switched. Note that at these fluences sputtering and amorphization are negligible, as a maximum of ∼2.5 × 10^–3^ displacements per surface atom are expected. Specifically, we observe a switching of the characteristic spectral weight distribution of the chiral CCDW states, which saturates at a fluence of ≳4 × 10^11^ ions/cm^2^ = 4000 ions/μm^2^. These data provide strong evidence that HCIs can be used as tools for electronic phase engineering of modern quantum materials.

We used a compact electron beam ion source (EBIS) from DIS Germany GmbH,^34,35^ integrated into the ASPHERE III setup at beamline P04 of PETRA III (DESY).^36^ The EBIS together with a compact Wien filter provided a charge-to-mass-selected continuous beam of ^129^Xe^8+^ ions with a kinetic energy of 22.5 keV. The beam was focused and steered by means of electrostatic lenses and deflectors onto the sample surface. An ion beam current of 12 pA was measured at the sample position without secondary electron compensation. The true ion beam current should therefore be 3–4 times smaller based on previous measurements of total electron yields under similar conditions.^37^ Since the secondary electron yield needed for a correction of the ion beam current is unknown for this particular setting, we herein use the *as-measured* current and fluence, which is then considered to be an upper bound of the true current. The irradiated area was determined to be an ellipse with axes *a* = 2.6 mm and *b* = 1.4 mm, with an area *A* = 11.3 mm^2^, and it was measured by scanning the edges of a metal plate of 5 × 5 mm^2^ through the beam in the horizontal and vertical directions while recording the fraction of the measured beam current. A current density of 12 pA/11.3 mm^2^ = 1.1 pA/mm^2^ was applied during the irradiation period. This value can be converted to ion fluence by considering that $1\;\mathrm{pA}\mathrm{=̂}10^{-12}\;C/s\mathrm{=̂}9.4\times 10^{6}$ particles with *q* = 8*e* per second. Including the measured beam area, we can estimate an ion flux of 9.4 × 10^6^ ions/11.3 mm^2^/s = 8.2 × 10^7^ ions/cm^2^/s. The angle of incidence of the ion beam was 60° with respect to the sample surface normal. Once the ion beam was aligned and positioned, a 1*T*-TaS_2_ sample was put at the target position instead of the metal plate.

The 1*T*-TaS_2_ sample was grown using the chemical vapor transport technique employing iodine as a transport agent.^38^ Once introduced into the ultrahigh-vacuum system, the bulk sample was mechanically cleaved under vacuum to prepare a fresh surface. The sample was quickly introduced into the main ARPES measurement chamber, which was at a pressure of <7 × 10^–11^ mbar, and the sample manipulator was cooled to 50 K to reach the CCDW phase.^39^ The sample was kept at 50 K for all of the measurements presented in this work.

A 400 eV soft X-ray beam with a constant circular polarization was focused to ∼15 × 15 μm^2^ on the sample surface. Photoelectrons were detected with a Scienta DA30-L hemispherical energy analyzer. A two-angle-energy map (full ARPES map) was recorded before the ion beam irradiation (ion beam blocked). According to this map the sample azimuthal angle was aligned such that the Γ point is in the center of the angle-dispersive direction of the spectrometer, which further reflects the *k*_*x*_ *′* axis (approximately 13° off ΓK) as given in Figure 1(a). Then ion irradiation was started, and a series of single-angle-energy maps (short ARPES maps along *k*_*x*_ *′*) were recorded. Each measurement was integrated for 30 s, corresponding to a fluence of 2.5 × 1 × 10^9^ ions/cm^2^ per map. After a total accumulated ion fluence of 8.1 × 10^11^ ions/cm^2^ (8100 ions/μm^2^), the ion beam was shut off. A final full ARPES map was then recorded using the same settings as for the first full ARPES map recorded prior to the ion irradiation.

Figure 1 shows *k*-space maps of the photoelectrons for two binding energies of *E* – *E*_F_ = −0.3 eV (a–c) and −0.8 eV (e–g), respectively, i.e., in the Ta 5d bands which show a Mott gap at these energies when the CCDW forms. Both energies are indicated as lines in the full energy spectrum shown in panel (d). Panels (a) and (e) show the photoemission intensity before the irradiation; panels (b) and (f) after the irradiation. Note that we show here the as-measured intensities (in arb. units) and that no normalization was applied, as the total intensity difference from the measurements before and after the irradiation amounts to only 0.08%. A reorientation of the photoemission intensity distribution is visible close to the indicated Γ*M* line. Panels (c) and (g) present the differences in intensity before and after HCI irradiation. A clear intensity redistribution is visible across the Γ*M* symmetry line, which corresponds to a mirror operation across the Γ*M* direction. Figure 1(h) presents line profiles indicated in (c) and (g), which demonstrate nearly perfect anticorrelation of the intensities before and after ion irradiation.

The relative change of photoemission intensity between the initial short ARPES map *I*_0_ (Figure 2(a)) and the maps obtained at two selected various ion fluences *I_n_* is shown in Figure 2(b,c). For a fluence of 3 × 10^11^ ions/cm^2^ (panel (b)), an intensity rearrangement (red to blue color) in the Ta 5d bands close to the Fermi edge near the Γ point (*k*_*x*_ *′* ∼ 0) can be seen, labeled as regions of interest (ROIs) 1 and 2. In addition, there are only minor changes in the band intensities at higher binding energy. Relative changes in the S 3p bands (with rather strong Ta 5d character^9,40,41^ ) around −4.5 eV (ROI3) become increasingly pronounced at higher fluences. Notably, the general band structure remains unchanged, and no increase in the isotropic photoemission background is visible. This is an indication that ion-induced amorphization is negligible at these fluences.

The photoemission intensity in the three ROIs (Ta 5d and S 3p bands; indicated in panels (a) and (b)) as a function of ion fluence is presented in Figure 2(d). For each ROI, a similar exponential fluence dependence can be observed: The photoemission intensity changes up to a characteristic fluence of (3.5–4.0) × 10^11^ ions/cm^2^ and saturates for larger fluences.

Figure 3 presents ARPES maps taken both before (a,c) and after (b,d) the full ion fluence was applied. The *k*-space cuts are along the paths Γ*KM*Γ and Γ*K′M*Γ as denoted in Figure 1, and band structure calculations from Ref. 42 are also shown. The turquoise and orange ellipses indicate specific photoelectron intensities in the Ta 5d bands, which appear below (↓) and above (↑) the dotted line at −0.45 eV. The intensity below the reference line at −0.45 eV switches from the *K′M* direction (a) before to *KM* (d) after ion irradiation. Similarly, the intensity above the reference line in (b) and (c) is redistributed from *KM* to *K′M*. Thus, the *E*–*k* maps in Figure 3(a) and (d) are equivalent, as are the maps in (c) and (b), respectively.

Since electronic bands are a direct consequence of the atomic lattice arrangement in solids,^43^ increasing disorder or amorphization would imply an increasingly isotropic photoemission (larger background in the ARPES maps).^44^ As this is not observed in our case, we assume that the Xe^8+^ ions mainly induce point defects or small defect clusters without excessive amorphization in the impact vicinity. Previous work also showed immunity of TaS_2_ devices to irradiation with high-energy protons^45^ and X-rays.^46^ HCI irradiation of transition-metal dichalcogenides and their heterostructures recently revealed that surface vacancy clusters result in atomically sharp crystalline boundaries without disorder in the remaining material.^30,47^

The process of surface modification by HCIs is the result of strong electronic excitations driven by ion neutralization processes.^48^ When a sufficient amount of surface electrons are promoted from valence bands to the conduction band in a nanometric volume, the surface atom binding becomes significantly weakened.^49^ Atoms can then either desorb from the surface, leaving vacancies,^29^ or rearrange in a different crystallographic phase once binding is restored.^50^ The CCDW phase of 1*T*-TaS_2_ is the result of strong electron–phonon coupling.^4^ This coupling is known to be the main link between electronic excitations from HCI impacts and lattice atom displacements.^51,52^

Figure 4 presents the schematic SoD structures for the two different chiral phases. Mirroring the structure (and rotating 120° for clarity here) leads to the alternate chiral phase. The CCDW is linked to the SoD superstructure, corresponding to a $\sqrt{13}\times \sqrt{13}R13.9^{{}^{\circ}}$ reconstruction. The regular and reconstructed Brillouin zones are shown in the lower panels of Figure 4. The chiral switching effectively causes the superstructure Brillouin zones to rotate by 27.8°. The intersections of the elliptical band pockets of the Ta 5d electrons with high symmetry points of the superstructure for different energies *E* – *E*_F_ are indicated by violet/red points. These intersections switch sites across the Γ*M* direction (cf. Figure 1(c) and (g)). As previously reported for the chiral phases,^7^ we detect a reorientation of the photoelectron intensity around these elliptical pockets in the same way, indicating a chiral switch.

Figure 2(e) illustrates a possible mechanism for ion-induced chiral switching. Since the complexity of a CCDW phase itself, the underlying electron–electron correlations, and the ion interaction with the surface involving electronic and nuclear degrees of freedoms across different time domains from femtoseconds to microseconds is currently surpassing the possibilities of comprehensive modeling, we want to suggest a possible plausible scenario as to what drives the observed chiral switch. As we observe a monotonic switching from the initial to the alternate chiral phase with a saturation of the handedness change for ∼3500–4000 ions/μm^2^, we can assume the following: An HCI impact extracts about 30 electrons from the impact site,^53–55^ which corresponds to the excess electron density of 30 SoDs.^56^ Additionally, several valence electrons will be promoted into the conduction band until most of the 417 eV potential energy of the ion is consumed. Strong electron–phonon coupling transfers the local excess energy in the electronic system to lattice heat. The energy barrier between the two chiral phases (<200 meV/Ta atom^13^) can then be overcome and the surface switches locally. The saturation fluence yields a switching cross section of ∼250 nm^2^, which contains 200 SoDs. Thus, we expect a fraction of ∼15% of SoD to be depleted of the excess electron to be sufficient for a chiral switch.

A switched island imposes a grain boundary between the chiral phases some nanometers from the impact site. At the impact site, the heavy Xe ion also causes lattice atom displacement as well as surface atom sputtering. Along with our scenario, we assume that these very localized lattice defects cause the chiral grain boundary to be pinned at its position. The switched chiral island can then not collapse by an inward-moving grain boundary, which would otherwise be assumed from the mobility of the grain boundaries above 30 K.^57^ It was shown recently by cryogenic STM and scanning tunneling spectroscopy^58^ that single defects can modify the Mott gap and bands close to *E*_F_ up to 5 nm from the defect site. Our somewhat larger defects might pin the chiral grain boundary up to a larger distance from the ion impact. In fact, a saturation ion fluence of ∼4000 ions/μm^2^ (cf. Figure 2(d)) indicates that a single ion switches the chirality in an area of approximately 250 nm^2^ (representing a chiral switching cross section). This corresponds to a circle with a radius of 9 nm where a defect could pin the grain boundary. Ultrafast low-energy electron diffraction (LEED) and optical switching of the chirality^59,60^ demonstrated a minimal structural correlation length of a chiral phase of ∼20 nm, which is similar to our determined characteristic switched grain size per incident ion. As the probability for the incoming ions to impinge on a nonswitched area decreases with increasing fluence, the increase in chiral fraction (relative change in Figure 2(d)) must therefore be less than linear.

Since we assume that the ion-induced defects locally stabilize the opposite chirality, the chiral switching might not be reversible at much larger fluences and without annealing the ion defects. We observe no evidence of back-switching of the chiral phase for a further increase in ion fluence up to ∼9 × 10^11^ ions/cm^2^, and the final state after the ion irradiation corresponds to a full switch and not a 50/50 handedness.

The question remains why each ion impact reverses the initial chirality and does not lead to a 50/50 probability for the initial and reversed chirality. To understand this, we recall that the surface chirality, probed by ARPES, is affected by the interlayer coupling in the bulk. It was recently shown by Zhao et al.^22^ that 1*T*-TaS_2_ favors homochirality across layers over homochirality in the surface plane. Thus, in our scenario, grain boundaries at the surface might form, depending on the interlayer interaction. Buried ion-induced defects (the penetration depth of 22.5 keV Xe under 60° in TaS_2_ is approximately 10 nm) break the symmetry between layers, leading to a local disorder between layers, which may then favor heterochirality at the surface and pin the surface chirality permanently.

The engineering of electronic bands in van der Waals materials is a promising pathway toward the tailoring of material properties at will. We showed that low fluences of HCIs are sufficient to enable band engineering and, in particular, are able to induce a chiral switch in the CCDW of 1*T*-TaS_2_. No indications of amorphization were found, and the overall small ion fluence of less than 1 ion per 1000 surface atoms indicates a negligible damage fraction at the surface. We suggest a plausible, comprehensive scenario in line with previous findings of other groups on HCIs and chiral switching, where the observed changes in the electronic band structure are explained by electronically mediated surface atom rearrangements. This gentle electronic surface modification is in stark contrast to ion sputtering by heavy ions, which becomes significant only at much higher fluences.

The key to induce a chiral switch on the nanoscale, by single-ion impacts, and its accumulation to a full chiral switch of the entire surface at larger ion fluences might be the use of HCIs. Using both simultaneously, the strong electronic excitation of the surface due to the elevated ion charge state and the high momentum transfer of the heavy ion seems to be needed to electronically switch the chiral phase and to stabilize the switched grain by point defects altering the interlayer interaction.

Future work should explore the importance of lattice disorder in the symmetry breaking of energetically degenerate surface states and specifically the role that ion beams can play in quantum material engineering.

## Figures and Tables

**Figure 5:**
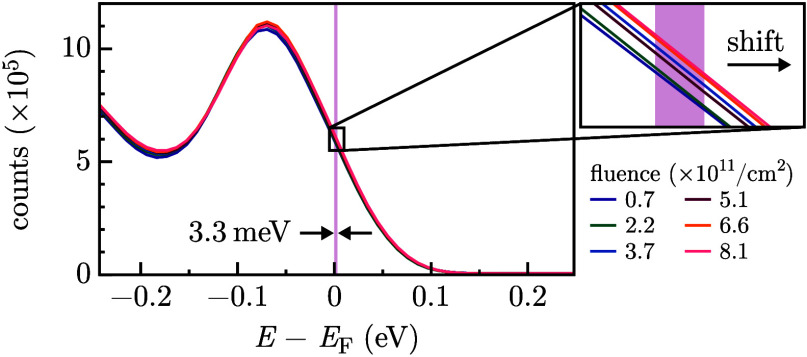
Fermi edge position for the different measurements. The *E* – *E*_*F*_ = 0 position is fixed at a 50% height between −0.1 and +0.1 eV for the first spectrum. This axis calibration is used for all other spectra. The 3.3 meV variation at 50% height is indicated as a red bar.

## Appendix

Energy Drift: During ion beam irradiation we recorded a series of ARPES spectra for 30 s each. Here, we integrated 60 spectra (1800 s) to obtain larger statistics. Figure 5 shows the position of the Fermi edge for each of the integrated spectra. The Fermi edge position varies by ∼3.3 meV throughout the measurement series, which is much smaller than the energy differences that we observe at other positions of the band structure (e.g., see Figure 2). We therefore conclude that no significant charge carrier doping occurs and that no significant drift in the spectrometer occurred. We consider the 3.3 meV variation as an estimate of the energy uncertainty in our measurement.
